# Prevalence of comorbid depression and associated factors among hospitalized patients with type 2 diabetes mellitus in Hunan, China

**DOI:** 10.1186/s12888-023-04657-4

**Published:** 2023-03-14

**Authors:** Rehanguli Maimaitituerxun, Wenhang Chen, Jingsha Xiang, Atipatsa C. Kaminga, Xin Yin Wu, Letao Chen, Jianzhou Yang, Aizhong Liu, Wenjie Dai

**Affiliations:** 1 Department of Epidemiology and Health Statistics, Xiangya School of Public Health, Central South University, Changsha, Hunan China; 2 Hunan Provincial Key Laboratory of Clinical Epidemiology, Changsha, Hunan China; 3 Department of Nephrology, Xiangya Hospital, Central South University, Changsha, Hunan China; 4 Human Resource Department, Central Hospital Affiliated to Shandong First Medical University, Jinan, Shandong China; 5 Department of Mathematics and Statistics, Mzuzu University, Mzuzu, Malawi; 6 Infection Control Center, Xiangya Hospital, Central South University, Changsha, Hunan China; 7 Department of Preventive Medicine, Changzhi Medical College, Changzhi, Shanxi China

**Keywords:** Depression, Type 2 diabetes mellitus, Hospitalized patients, Associated factors, Hunan

## Abstract

**Background:**

Depression and diabetes are major health challenges, with heavy economic social burden, and comorbid depression in diabetes could lead to a wide range of poor health outcomes. Although many descriptive studies have highlighted the prevalence of comorbid depression and its associated factors, the situation in Hunan, China, remains unclear. Therefore, this study aimed to identify the prevalence of comorbid depression and associated factors among hospitalized type 2 diabetes mellitus (T2DM) patients in Hunan, China.

**Methods:**

This cross-sectional study involved 496 patients with T2DM who were referred to the endocrinology inpatient department of Xiangya Hospital affiliated to Central South University, Hunan. Participants’ data on socio-demographic status, lifestyle factors, T2DM-related characteristics, and social support were collected. Depression was evaluated using the Hospital Anxiety and Depression Scale-depression subscale. All statistical analyses were conducted using the R software version 4.2.1.

**Results:**

The prevalence of comorbid depression among hospitalized T2DM patients in Hunan was 27.22% (95% Confidence Interval [CI]: 23.3–31.1%). Individuals with depression differed significantly from those without depression in age, educational level, per capita monthly household income, current work status, current smoking status, current drinking status, regular physical activity, duration of diabetes, hypertension, chronic kidney disease, stroke, fatty liver, diabetic nephropathy, diabetic retinopathy, insulin use, HbA1c, and social support. A multivariable logistic regression model showed that insulin users (adjusted OR = 1.86, 95% CI: 1.02–3.42) had a higher risk of depression, while those with regular physical activity (adjusted OR = 0.48, 95% CI: 0.30–0.77) or greater social support (adjusted OR = 0.20, 95% CI: 0.11–0.34) had a lower risk of depression. The area under the curve of the receiver operator characteristic based on this model was 0.741 with a sensitivity of 0.785 and specificity of 0.615.

**Conclusions:**

Depression was moderately prevalent among hospitalized T2DM patients in Hunan, China. Insulin treatment strategies, regular physical activity, and social support were significantly independently associated with depression, and the multivariable model based on these three factors demonstrated good predictivity, which could be applied in clinical practice.

## Background

Diabetes is one of the fastest growing chronic diseases across the globe, causing microvascular and macrovascular complications and reduced life expectancy [1]. According to the latest data released by the International Diabetes Federation, the global prevalence of diabetes was 10.5% in 2021 and is expected to rise to 12.2% in 2045; the number of people affected by the disease is estimated to increase to 783.2 million in 2045 from 536.6 million in 2021 [2]. China was ranked first by the number of adults with diabetes in 2021 (140.9 million) and is expected to retain its position in 2045 (174.4 million) [2]. According to a nationally-representative cross-sectional study conducted by the Chinese Center for Disease Control and Prevention, the overall standardized prevalence estimation of diabetes increased significantly from 10.9% (95% Confidence Interval [CI]: 10.4–11.5%) in 2013 to 12.4% (95% CI: 11.8–13.0%) in 2018 [3]. Type 2 diabetes mellitus (T2DM) accounts for the majority of diabetes cases, with its prevalence increasing from 3.7% (95% CI: 3.6–3.8%) in 2008 to 6.6% (95% CI: 6.4–6.7%) in 2017, in Beijing. However, the annual rate of increase slowed from 18.1% (95% CI: 14.4–22.0%) to 1.5% (95% CI: 0.8–2.2%) before and after 2011, respectively [4].

Depression, characterized by persistent sadness and a lack of interest or pleasure in previously rewarding or enjoyable activities [5], is one of the most common mental disorders that coexist with T2DM patients [6–8]. Comorbid depression in T2DM patients significantly worsens prognosis and raises mortality rates by increasing the incidence of microvascular and macrovascular complications, as well as reducing treatment compliance and self-care ability. This could ultimately lead to impaired glycemic control and poor quality of life [9–11]. Additionally, compared with those with only diabetes, comorbid depression combined with diabetes could lead to higher healthcare costs and a greater socioeconomic burden [12, 13]. Therefore, understanding the prevalence of comorbid depression and associated factors among T2DM patients is critical not only for appropriate allocation of psychological intervention resources for healthcare providers but also for the facilitation of early identification of those with a heightened risk of depression.

Globally, numerous studies have addressed the issue of comorbid depression in T2DM patients. An updated systematic review and meta-analysis showed that the pooled prevalence of comorbid depression among T2DM patients was 28% globally, 24% in Europe, 27% in Africa, 29% in Australia, and 32% in Asia [14]. Liu et al. [15] found that the pooled prevalence of comorbid depression among T2DM patients in China was 25.9% (95% CI: 20.6–31.6%), with the figure being higher in females, participants aged ≥60 years, those with a primary school or lower education, individuals with a duration of T2DM ≥10 years, participants with diabetic complications, insulin users and participants living alone, and being lower in those with current alcohol use. However, no study has reported the prevalence of comorbid depression and associated factors among hospitalized patients with T2DM in Hunan, which is located in central China and middle reaches of the Yangtze River. The province covers an area of 211.8 thousand square kilometers, with a registered population of 73 million and a permanent population of 69.18 million in 2020. The present study aimed to identify the prevalence of comorbid depression and associated factors among hospitalized T2DM patients in Hunan, using a cross-sectional study design to comprehensively assess the role of socio-demographic status, lifestyle factors, T2DM-related characteristics, and social support in comorbid depression among T2DM patients.

## Methods

### Study design and participants

This cross-sectional study was conducted in Hunan, China. All individuals referred to the endocrinology inpatient department of Xiangya Hospital affiliated to Central South University—a top-level general hospital located in the capital city of Hunan Province (Changsha), China—between March and December 2021, with T2DM confirmed by the endocrinologist, and aged ≥40 years were consecutively invited to participate in this study. Those with dementia or who could not speak Mandarin were excluded. The sample size of this study was 496, which was more than the minimum sample required of 385 as determined based on the sample size formula for categorical outcome (proportion) in cross-sectional studies (N = Z^2^p(1-p)/d^2^) [16] using these assumptions: Z = 1.96, *p* = 20.6% (the lower limit of the 95% CI of the pooled prevalence of comorbid depression among T2DM patients in China reported by a previous meta-analysis) [15], and d = 0.2p.

### Procedures

Data on socio-demographic status, lifestyle factors, social support, and depression were collected by investigators who underwent unified training and had at least a bachelor’s degree in medicine. Information such as body mass index (BMI), duration of diabetes, family history of diabetes, diabetic complications and comorbidities, and insulin treatment strategies was obtained from electronic medical records. HbA1c was measured as part of routine inpatient visits using an ARKRAY automatic glycohemoglobin analyzer (ARKRAY Factory, Shanghai, China) on the ADAMS A1c HA-8180 system.

### Outcome of interest

The primary outcome of this study was the prevalence of comorbid depression. The Hospital Anxiety and Depression Scale-depression subscale (HADS-D) was used to evaluate depressive symptoms. The HADS-D, developed by Zigmond and Snaith, is a 7-item self-report scale with 4 response alternatives from 0 to 3 [17]. Its total score ranges between 0 and 21 with a score of ≥8 recommended as the cut off level for depression [18, 19]. Specifically, those with a total score of ≥8 were categorized into depression group, and those with a total score of < 8 were categorized into non-depression group. The HADS-D had satisfactory reliability and validity in the Chinese population. The concurrent validity of the HADS-D compared to the mental component summary of the Chinese version of Medical Outcomes Study 12-item Short Form (C-SF-12) (version 2) was described between − 0.53 and − 0.51 [20], and the Cronbach’s α coefficients ranged from 0.81 to 0.87 [20–22]. In the current study, the Cronbach’s α coefficient of HADS-D was 0.837.

### Independent variables

The independent variables of this study were socio-demographic status (age, sex, ethnicity, marital status, educational level, household income, living status, and current work status), lifestyle factors (current smoking and drinking status, and regular physical activity), T2DM related characteristics (BMI, duration of diabetes, family history of diabetes, diabetic complications and comorbidities, HbA1c, and insulin treatment strategies), and social support.

Current smoking was defined as smoking greater than or equal to 1 cigarette in the past 30 days, current drinking was defined as having at least one drink of any alcoholic beverage in the past 30 days, and regular physical activity was defined as the performance of at least one activity, such as walking, square dancing, and cycling, for at least 30 minutes per day in the past 30 days. T2DM related characteristics were collected from the electronic medical records, and BMI was categorized into < 24 and ≥ 24 based on Chinese Guidelines for Medical Nutrition Treatment of Overweight/Obesity (2021 edition) [23]. The duration of diabetes was categorized into < 10 years and ≥ 10 years based on the findings of previous studies [24–26], and HbA1c was categorized into ≤7 and > 7% according to the Clinical Guidelines for Prevention and Treatment of Type 2 Diabetes Mellitus in older adults in China (2022 edition) [27].

Social support was measured using the Social Support Rating Scale (SSRS) [28], which is a 10-item scale with three dimensions—subjective support (4 items), objective support (3 items), and support utilization (3 items). The total score ranges from 12 to 66, with higher scores suggesting greater social support. A total score of > 44 (≤44) was regarded as high (low) social support. The SSRS has been widely used and is well validated in Chinese populations [29, 30], and in the current study, the Cronbach’s α coefficient of SSRS was 0.788.

### Statistical analyses

Continuous variables distributed normally or abnormally were described by mean ± standard deviation (SD) or median and interquartile range (IQR). Categorical variables were described by frequency (n) and proportion (%).

The difference between the depression and non-depression groups by each independent variable was examined using the chi-square test. The contribution of each independent variable to the outcome variable (depression) was quantified by crude OR and its corresponding 95% CI using univariable logistic regression analyses. Independent variables that differed significantly between the depression and non-depression groups were entered into the multivariable logistic regression model, from which the contribution of each independent variable was quantified by adjusted OR (aOR) and its corresponding 95% CI. Finally, the receiver operator characteristic (ROC) curve based on the multivariable logistic regression model was drawn to evaluate the predictive value of the model. All statistical analyses were two-sided at the 5% significant level and were conducted in the R software version 4.2.1 (https://www.r-project.org/).

## Results

### Characteristics of the study participants

The mean age of the study population was 59.57 ± 9.92 with a range of 40 to 96. Among the 496 participants, 284 (57.26%) were men, 469 (94.56%) were Han Ethnicity, 447 (90.12%) were married, and 217 (43.75%) attended high school or above. Regarding lifestyle, 83 (16.73%) were current smokers, 56 (11.29%) were current drinkers, and 320 (64.52%) performed regular physical activity. The mean duration of diabetes was 11.21 ± 7.75 years, with the majority (54.84%) having duration of diabetes ≥10 years. Furthermore, 314 (63.31%), 138 (27.82%), 92 (18.55%), 165 (33.27%), 71 (14.32%), and 117 (23.60%) of the participants had hypertension, hyperlipidemia, coronary heart disease, chronic kidney disease, stroke, and fatty liver, respectively, whereas 262 (52.82%), 226 (45.57%), 49 (9.88%), 379 (76.41%), and 235 (47.38%) participants had diabetic nephropathy, diabetic retinopathy, diabetic foot, diabetic peripheral neuropathy, and diabetic peripheral vascular disease, respectively. The mean total score of SSRS was 42.14 ± 7.55, and based on the cutoff value of 44, 289 (58.27%) and 207 (41.73%) were categorized as having low and high social support, respectively (Tables 1 and 2).

**Table 1:** Socio-demographic and lifestyle factors of the study participants (*n* = 496)

Independent variable	Category	Frequency (n)	Proportion (%)
Age	40–59 years	271	54.63
	≥60 years	225	45.36
Sex	Male	284	57.26
	Female	212	42.74
Ethnicity	Han	469	94.56
	Minority	27	5.44
Marital status	Married	447	90.12
	Unmarried	49	9.88
Educational level	Middle school or below	279	56.25
	High school or above	217	43.75
Per capita monthly household income	≤5000 yuan	341	68.75
	> 5000 yuan	155	31.25
Living alone	Yes	30	6.05
	No	466	93.95
Current work status	Employed	145	29.23
	Not employed	351	70.77
Current smoking status	No	413	83.27
	Yes	83	16.73
Current drinking status	No	440	88.71
	Yes	56	11.29
Regular physical activity	No	176	35.48
	Yes	320	64.52

**Table 2:** T2DM-related characteristics of the study participants (*n* = 496)

Independent variable	Category	Frequency (n)	Proportion (%)
BMI	< 24	269	54.23
	≥24	227	45.77
Duration of diabetes	< 10 years	224	45.16
	≥10 years	272	54.84
Family history of diabetes	No	271	54.64
	Yes	225	45.36
Hypertension	No	182	36.69
	Yes	314	63.31
Hyperlipidemia	No	358	72.18
	Yes	138	27.82
Coronary heart disease	No	404	81.45
	Yes	92	18.55
Chronic kidney disease	No	331	66.73
	Yes	165	33.27
Stroke	No	425	85.69
	Yes	71	14.32
Fatty liver	No	379	76.41
	Yes	117	23.60
Diabetic nephropathy	No	234	47.18
	Yes	262	52.82
Diabetic retinopathy	No	270	54.44
	Yes	226	45.57
Diabetic foot	No	447	90.12
	Yes	49	9.88
Diabetic peripheral neuropathy	No	117	23.59
	Yes	379	76.41
Diabetic peripheral vascular disease	No	261	52.62
	Yes	235	47.38
Insulin use	No	122	24.60
	Yes	374	75.40
HbA1c	≤7%	113	22.78
	> 7%	383	77.22
Social support	Low	289	58.27
	High	207	41.73

BMI, body mass index

### Prevalence of comorbid depression

The median (IQR) of the total score of HADS-D was 5.00 (6.00), and according to the cutoff value of 8, 135 and 361 participants were categorized into depression group and non-depression group, respectively. The prevalence of comorbid depression among hospitalized T2DM patients in Hunan was 27.22% (95% CI: 23.3–31.1%).

### Univariable analyses of factors associated with comorbid depression

Tables 3 and 4 show the results of univariable associations between the independent variables and comorbid depression. The depression and non-depression groups differed significantly in relation to age, educational level, per capita monthly household income, current work status, current smoking status, current drinking status, regular physical activity, duration of diabetes, hypertension, chronic kidney disease, stroke, fatty liver, diabetic nephropathy, diabetic retinopathy, insulin use, HbA1c, and social support (*P <* 0.05).

**Table 3:** Univariable analyses for the associations of socio-demographic and lifestyle factors with comorbid depression

Independent variable	Category	Depression group (*n* = 135, %)	Non-depression group (*n* = 361, %)	Crude OR (95% CI)	*P* value
Age	40–59 years	58 (42.96)	213 (59.00)	1	0.001^**^
	≥60 years	77 (57.04)	148 (41.00)	1.91 (1.28–2.85)	
Sex	Male	68 (50.37)	216 (59.83)	1	0.058
	Female	67 (49.63)	145 (40.17)	1.47 (0.99–2.18)	
Ethnicity	Han	130 (96.30)	339 (93.91)	1	0.296
	Minority	5 (3.70)	22 (6.09)	0.59 (0.22–1.60)	
Marital status	Married	116 (85.9)	331 (91.69)	1	0.056
	Unmarried	19 (14.07)	30 (8.31)	1.81 (0.98–3.33)	
Educational level	Middle school or below	96 (71.11)	183 (50.69)	1	< 0.001^***^
	High school or above	39 (28.89)	178 (49.31)	0.42 (0.27–0.64)	
Per capita monthly household income	≤5000 yuan	114 (84.44)	227 (62.88)	1	< 0.001^***^
	> 5000 yuan	21 (15.56)	134 (37.12)	0.31 (0.19–0.52)	
Living alone	Yes	12 (8.89)	18 (4.99)	1	0.105
	No	123 (91.11)	343 (95.01)	0.54 (0.25–1.25)	
Current work status	Employed	18 (13.33)	127 (35.18)	1	< 0.001^***^
	Not employed	117 (86.67)	234 (64.82)	3.53 (2.05–6.06)	
Current smoking status	No	120 (88.89)	293 (81.16)	1	0.040^*^
	Yes	15 (11.11)	68 (18.84)	0.54 (0.30–0.98)	
Current drinking status	No	126 (93.33)	314 (86.98)	1	0.047^*^
	Yes	9 (6.67)	47 (13.02)	0.48 (0.23–0.99)	
Regular physical activity	No	68 (50.37)	108 (29.92)	1	< 0.001^***^
	Yes	67 (49.63)	253 (70.09)	0.43 (0.23–0.63)	

^*^ *P <* 0.05; ^**^ *P *< 0.01; ^***^ *P *< 0.001

**Table 4:** Univariable analyses for the association of T2DM related characteristics with comorbid depression

Independent variable	Category	Depression group (*n* = 135, %)	Non-depression group (*n* = 361, %)	Crude OR (95% CI)	*P* value
BMI	< 24	74 (54.81)	195 (54.02)	1	0.874
	≥24	61 (45.19)	166 (45.98)	0.97 (0.65–1.44)	
Duration of diabetes	< 10 years	50 (37.04)	174 (48.20)	1	0.002^**^
	≥10 years	85 (62.96)	187 (51.80)	1.96 (1.27–3.04)	
Family history of diabetes	No	76 (56.30)	195 (54.02)	1	0.650
	Yes	59 (43.70)	166 (45.98)	0.91 (0.61–1.36)	
Hypertension	No	100 (74.07)	214 (59.28)	1	0.002^**^
	Yes	35 (25.93)	147 (40.72)	1.96 (1.27–3.04)	
Hyperlipidemia	No	34 (25.19)	104 (28.81)	1	0.423
	Yes	101 (74.81)	257 (71.19)	0.83 (0.53–1.31)	
Coronary heart disease	No	32 (23.70)	60 (16.62)	1	0.071
	Yes	103 (76.30)	301 (83.38)	1.56 (0.96–2.53)	
Chronic kidney disease	No	59 (43.70)	106 (29.36)	1	0.003^**^
	Yes	76 (56.30)	255 (70.64)	1.87 (1.24–2.81)	
Stroke	No	29 (21.48)	42 (11.63)	1	0.005^**^
	Yes	106 (78.52)	319 (88.37)	2.08 (1.23–3.50)	
Fatty liver	No	22 (16.30)	95 (26.32)	1	0.019^*^
	Yes	113 (83.70)	266 (73.68)	0.55 (0.33–0.91)	
Diabetic nephropathy	No	90 (66.67)	172 (47.65)	1	< 0.001^***^
	Yes	45 (33.33)	189 (52.35)	2.20 (1.45–3.32)	
Diabetic retinopathy	No	77 (57.04)	149 (41.27)	1	0.002^**^
	Yes	58 (42.96)	212 (58.73)	1.89 (1.27–2.82)	
Diabetic foot	No	16 (11.85)	33 (9.14)	1	0.368
	Yes	119 (88.15)	328 (90.86)	1.34 (0.71–2.52)	
Diabetic peripheral neuropathy	No	28 (20.74)	89 (24.65)	1	0.361
	Yes	107 (79.26)	272 (75.35)	1.25 (0.71–2.52)	
Diabetic peripheral vascular disease	No	71 (52.59)	190 (52.63)	1	0.994
	Yes	64 (47.41)	171 (47.37)	1.00 (0.67–1.49)	
Insulin use	No	21 (15.56)	101 (27.98)	1	0.004^**^
	Yes	114 (84.44)	260 (72.02)	2.11 (1.25–3.54)	
HbA1c	≤7%	64 (47.41)	131 (36.29)	1	0.014^*^
	> 7%	71 (52.59)	230 (63.71)	0.24 (0.05–0.44)	
Social support	Low	114 (84.44)	175 (48.48)	1	< 0.001^***^
	High	21 (15.56)	186 (51.52)	0.17 (0.10–0.29)	

^*^ *P <* 0.05; ^**^ *P *< 0.01; ^***^ *P *< 0.001; BMI, body mass index

### Multivariable analyses of factors associated with comorbid depression

**Fig. 1:**
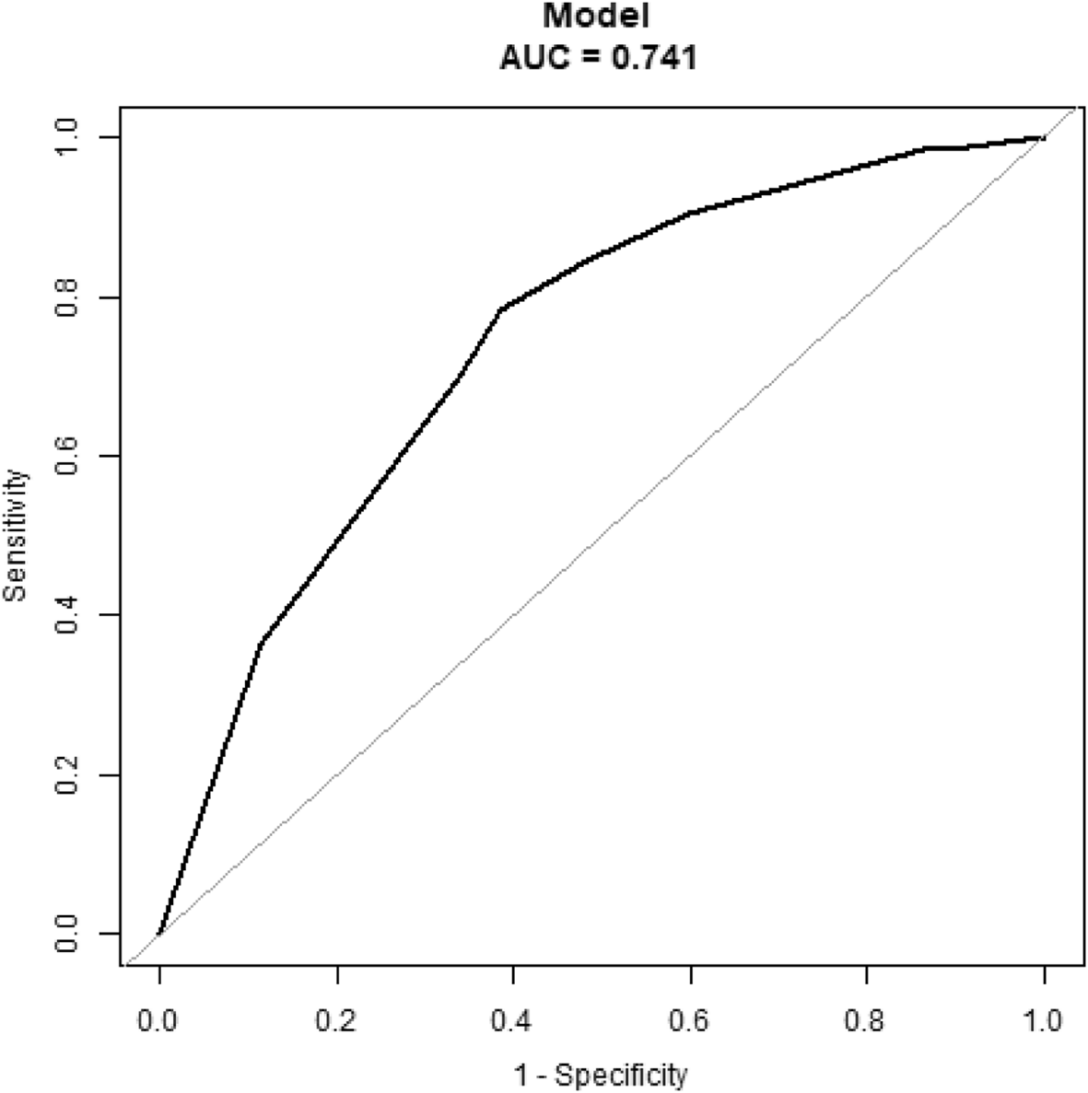
The ROC curve based on the multivariable logistic regression model

Table 5 shows the results of the multivariable logistic regression model on factors associated with comorbid depression among hospitalized T2DM patients in Hunan. Insulin users (aOR = 1.86, 95% CI: 1.02–3.42, *P* = 0.044) were at an increased risk of comorbid depression. Those performing regular physical activity (aOR = 0.48, 95% CI: 0.30–0.77, *P* = 0.002) or having higher social support (aOR = 0.20, 95% CI: 0.11–0.34, *P* < 0.001) had a reduced risk of comorbid depression. The ROC curve based on the multivariable logistic regression model including the factors of insulin treatment strategies, regular physical activity, and social support is shown in Fig. 1. The area under the curve (AUC) was 0.741 with a sensitivity of 0.785 and a specificity of 0.615.

**Table 5:** Multivariable logistic regression analysis on factors associated with comorbid depression

Independent variable	Description	*B*	*SE*	Adjusted OR (95% CI)	*P* value
Age	40–59 years Vs ≥60 years	−0.518	0.282	1.27 (0.74–2.18)	0.379
Educational level	Middle school or below Vs High school or above	−0.518	0.282	0.59 (0.34–1.03)	0.064
Per capita monthly household income	≤5000 yuan Vs > 5000 yuan	−0.514	0.331	0.60 (0.31–1.14)	0.120
Current work status	Employed Vs Not employed	0.498	0.345	1.63 (0.83–3.21)	0.155
Current smoking status	No Vs Yes	−0.501	0.384	0.61 (0.29–1.30)	0.204
Current drinking status	No Vs Yes	0.268	0.49	1.31 (0.50–3.41)	0.585
Regular physical activity	No Vs Yes	−0.720	0.239	0.48 (0.30–0.77)	0.002^**^
Duration of diabetes	< 10 years Vs ≥10 years	0.089	0.156	1.24 (0.59–2.59)	0.565
Hypertension	No Vs Yes	0.222	0.279	1.24 (0.72–2.15)	0.437
Chronic kidney disease	No Vs Yes	0.287	0.31	1.33 (0.72–2.44)	0.364
Stroke	No Vs Yes	0.570	0.319	1.75 (0.94–3.28)	0.079
Fatty liver	No Vs Yes	−0.246	0.311	0.79 (0.43–1.45)	0.447
Diabetic nephropathy	No Vs Yes	0.108	0.316	1.11 (0.6–2.07)	0.734
Diabetic retinopathy	No Vs Yes	0.426	0.255	1.54 (0.93–2.54)	0.091
Insulin use	No Vs Yes	0.613	0.308	1.86 (1.02–3.42)	0.044^*^
HbA1c	≤7% Vs > 7%	−0.427	0.267	0.65 (0.38–1.10)	0.108
Social support	Low Vs High	−1.624	0.28	0.20 (0.11–0.34)	< 0.001^***^

^*^ *P <* 0.05; ^**^ *P *< 0.01; ^***^ *P *< 0.001

## Discussion

Compared with healthy controls, T2DM patients had a two-fold risk of developing depression [6], with comorbid depression in T2DM potentially leading to a wide range of poor health outcomes [31–33]. The present study was aimed at identifying the prevalence of comorbid depression and associated factors among hospitalized T2DM patients in Hunan. Few studies have evaluated the prevalence of comorbid depression among hospitalized patients exclusively. These limited studies showed that the prevalence was 49.2% (70/142) in Pakistan [34], 33.1% (47/142) in Morocco [35], 53.8% (85/160) in Saudi Arabia [36], and 23.2% (50/216) in Vietnam [37]. The prevalence of comorbid depression among hospitalized T2DM patients in Hunan, found in the present study (27.22%, 135/496) was lower than that in Pakistan and Saudi Arabia and almost comparable with that in Vietnam and Morocco. The differences in the economic development level, cultural background, instruments used to assess depression, and sample characteristics in diabetes severity may account for the prevalence differences observed for different countries. Given the negative effects brought by the coexistence of depression and T2DM, it is suggested that in addition to regular blood glucose monitoring, routine screening of depression should be conducted among hospitalized T2DM patients by well-trained healthcare professionals in clinical practice in Hunan. Furthermore, timely and effective support such as psychosocial intervention and cognitive behavioral therapy should be implemented for those with comorbid depression [38, 39].

Socio-demographic characteristics such as sex, age, educational level, and income were found to be associated with comorbid depression among T2DM patients in many previous studies [34, 37, 40–46]. In a recent systematic review and meta-analysis by Liu et al. [15], the pooled prevalence of comorbid depression among population-based T2DM patients in China was found to be higher in females, participants aged ≥60, those with a primary school or lower education, and individuals living alone. However, some studies showed different results. For example, Tran et al. [37] found that sex was not associated with comorbid depression among hospitalized T2DM patients, whereas Huang et al. [42] found that sex was independently associated with the prevalence and incidence of comorbid depression among diabetes patients in Taiwan. Similarly, Khan et al. [34] found that higher scores of depression were significantly associated with sex and age. This study showed that the prevalence of comorbid depression among T2DM patients was univariately but not independently associated with age, educational level, per capita monthly household income, and current work status. Therefore, it is imperative to conduct more studies with a larger sample of hospitalized T2DM patients to identify the association between socio-demographic characteristics and comorbid depression among hospitalized T2DM patients in China.

Regular physical activity is an important component of treatment strategies for diabetes patients, and its association with depression has been well-established among the general population [47, 48]. In diabetic populations, Mendes et al. [49] found that for older outpatients with diabetes, those with non-adherence to physical activity showed more depressive symptoms. Additionally, Ahola et al. [50] found that, after adjustments, more leisure-time physical activity was associated with more depressive symptoms in adult individuals with type 1 diabetes mellitus. Furthermore, a study using data from the Korea National Health and Nutritional Examination Survey [51] found that moderate intensity physical activity at work and during leisure time affected depression. Similarly, the present study found that regular physical activity in hospitalized T2DM patients was associated with a lower risk of depression after adjustments. Therefore, it is highly recommended for healthcare professionals in China to promote healthy lifestyles, including regular physical activity, among hospitalized T2DM patients to help manage depressive symptoms. Additionally, as family support is crucial for adherence to physical activity, efforts should be made by family members to encourage increased physical activity among hospitalized T2DM patients.

Diabetic neuropathy affects up to 50% of diabetes patients and is a major cause of morbidity and increased mortality. Its clinical manifestations include painful neuropathic symptoms and insensitivity, which increases the risk of burns, injuries, and foot ulceration [52]. The presence of diabetic neuropathy could worsen quality of life and induce changes in social and family roles, thus increasing the risk of depression [53]. A previous meta-analysis with 13 eligible studies involving 3898 individuals confirmed the association between diabetic neuropathy and depression among T2DM patients [54]. However, the present study found that diabetic retinopathy was associated with depression in the univariable analyses but not in the fully adjusted multivariable model. It is worth noting here that in the multivariable model, the contribution of diabetic retinopathy to depression has reached the margin of statistical significance (aOR = 1.54, 95%CI: 0.93–2.54, *P* = 0.091). Therefore, caution should be taken when interpreting this association.

For T2DM patients, insulin is the cornerstone of treatment for lowering glucose and HbA1c concentrations [55]. Although the optimal timing and indications for insulin therapy remain controversial, most of the patients inevitably require insulin therapy to attain adequate glycemic control in the natural history of T2DM [56, 57]. In the present study, 75.40% of the participants were treated with insulin, and this group had a 1.86-fold risk of developing depression compared with their counterparts. This is consistent with a previous meta-analysis that included 12 eligible studies [58]. Compared with those who were not on insulin treatment, those on insulin treatment were more likely to have advanced T2DM with less endogenous insulin, increasing their susceptibility to metabolic dysregulation; hence, they were more vulnerable to develop depression [59]. Therefore, those on insulin treatment may need more regular check-ups for depression in clinical practice.

Social support is a psychosocial element that influences people by providing them with emotional, informational, companionship, and financial support to increase their adherence to diabetes treatment and management guidelines [60]. The protective role of social support against depression has been identified in different populations in secondary data analyses [61–63]. Accordingly, Azmiardi et al. [64] conducted a systematic review and meta-analysis of 11 eligible studies involving a total of 3151 individuals and found that T2DM patients with lower social support had a two-fold risk of depression than those with greater social support. Similarly, this study found that higher social support was associated with a lower risk of depression after adjustments. These findings, therefore, underscore the importance of social support in the control and management of depression among hospitalized T2DM patients in China. Additionally, to build a systematic approach to reduce the burden of comorbid depression in T2DM, further studies on the mechanisms underlying this relationship are required.

This study found that nearly one fourth of hospitalized T2DM patients suffered from depression in Hunan, China. Considering the linkage between comorbid depression and subsequent poor health outcomes, routine screening for depression among hospitalized T2DM patients in this area is highly recommended. Insulin treatment strategies, regular physical activity, and social support were independently associated with depression. Therefore, the risk of comorbid depression among hospitalized T2DM patients might be reduced through enhancing physical activity and offering more social support in clinical practice, and those on insulin treatment should be paid special attention for preventing comorbid depression.

Some limitations should be considered when interpreting the findings of the present study. This was a cross-sectional study, implying that the associations of insulin treatment strategies, regular physical activity, and social support with comorbid depression might be bidirectional. Based on the considerations of recall bias, this study collected information on current smoking and drinking instead of the amount and frequency of smoking and drinking, which might play an important role in the presence of depression. Therefore, future prospective studies are needed to elucidate the causal relationships. Additionally, this study was hospital-based with a single-centre of participants aged ≥40 in Hunan, and the mean duration of diabetes of the participants was 11.21 ± 7.75 years with the majority having duration of diabetes ≥10 years. This may preclude the possibility of identifying the association between newly-diagnosed diabetes and depression, and whether the findings can be generalized into all T2DM patients in Hunan remains unclear. Therefore, future studies with larger, more representative samples of T2DM patients are needed.

## Conclusions

Depression is moderately prevalent with 27.22% of hospitalized T2DM patients suffering from depression in Hunan. Routine screening for depression among hospitalized T2DM patients in this area is highly recommended. Participants undergoing insulin treatment have a higher risk of comorbid depression, and those with regular physical activity or higher social support were at a lower risk of comorbid depression. The multivariable model based on the foregoing factors showed good predictivity, suggesting that it may be useful in clinical practice.

## Data Availability

The datasets generated and/or analyzed during the present study are not publicly available but are available from the corresponding author Wenjie Dai (Email: m18673965791@163.com) on reasonable request.

## Abbreviations

AUC: Area under the curve
aOR: Adjusted odds ratio
BMI: Body mass index
CI: Confidence Interval
HADS-D: The Hospital Anxiety and Depression Scale-depression subscale
IQR: Interquartile range
OR: Odds ratio
ROC: Receiver operator characteristic
SD: Standard deviation
SSRS: The Social Support Rating Scale
T2DM: Type 2 diabetes mellitus
